# Hepatitis B virus mutations, expression quantitative trait loci for PTPN12, and their interactions in hepatocellular carcinoma

**DOI:** 10.1002/cam4.712

**Published:** 2016-04-14

**Authors:** Ci Song, Yao Liu, Lu Xu, Juan Wen, Deke Jiang, Jianguo Chen, Xiangjun Zhai, Zhibin Hu, Li Liu, Jibin Liu

**Affiliations:** ^1^ Department of EpidemiologyCollaborative Innovation Center For Cancer Personalized MedicineSchool of Public HealthNanjing Medical UniversityNanjing211166China; ^2^ Jiangsu Key Lab of Cancer BiomarkersPrevention and TreatmentCollaborative Innovation Center of Cancer MedicineNanjing Medical UniversityNanjing211166China; ^3^ Pathology Center and Department of PathologySoochow UniversitySuzhouChina; ^4^ Nanjing Maternity and Child Health Care InstituteNanjing Maternity and Child Health Care Hospital Affiliated with Nanjing Medical UniversityNanjingChina; ^5^ State Key Laboratory of Genetic EngineeringCollaborative Innovation Center for Genetics and DevelopmentSchool of Life SciencesFudan UniversityShanghaiChina; ^6^ Qidong Liver Cancer InstituteQidongChina; ^7^ Department of Infection DiseasesJiangsu Province Center for Disease Prevention and ControlNanjingChina; ^8^ Digestive Endoscopy Centerthe First Affiliated Hospital of Nanjing Medical UniversityNanjingChina; ^9^ Department of Hepatobiliary SurgeryNantong Tumor HospitalNantongChina

**Keywords:** eQTL, HBV mutations, HCC, interaction, *PTPN12*, susceptibility

## Abstract

Previously we identified that HBV(Hepatitis B virus) sequence variation, which may interact with host human leukocyte antigen (*HLA*) genetic variation, could influence host risk of hepatocellular carcinoma (HCC). More HBV‐host interactions need to be identified. Protein tyrosine phosphatase nonreceptor type 12 (PTPN12), serves as an antagonist to tyrosine kinase signaling, may play integral roles in immune response against HBV infection and the development of HCC. Rs11485985 was an expression quantitative trait loci (eQTL) for *PTPN12* by bioinformatics analyses. In this study, we genotyped the *PTPN12* eQTL and sequenced the HBV region EnhII/BCP/PC in a case–control cohort including 1507 HBV‐related HCC cases and 1560 HBV persistent carriers as controls. The variant genotype GG of rs11489585 increased HCC risk compared to the HBV persistent carriers (adjusted OR = 2.03, 95% confidence interval [CIs] = 1.30–3.18). We also detected borderline significant associations of *PTPN12* eQTL rs11489585 with HBV mutations (*P* = 0.05 for G1799C). Taken together, *PTPN12* may influence HCC risk accompanied by HBV mutations.

## Introduction

Hepatocellular carcinoma (HCC) is the second leading cause of cancer‐related deaths worldwide. In particular, China accounts for almost 50% of the world's HCC cases 1. HCC may be affected by several known environmental factors, including hepatitis viruses, alcohol, cigarette smoking, and others. In addition, individual genetic predisposition may play a role in the risk of HCC 2.

In the last decades, 10 HBV genotypes (genotypes A–J) have been orderly identified by a sequence divergence >8% in the entire HBV genome 3, 4, 5. HBV genotypes have distinct geographic distributions, while HCC incidence also varies in different regions of the world 6. Previous study also revealed that some HBV genotypes may be more carcinogenic 7. Four genes overlapped within the HBV genome. The precore/core encodes hepatitis B e surface antigen (HBeAg), which is used clinically as an indicator of active viral replication and is associated with an increased risk of HCC 8, 9. The basal core promoter, which is regulated by the enhancer II, controls the transcription of precore mRNA 10. Thus, HBV sequence mutations with potential regulatory role may influence the outcome of HBV infection. However, on account of the small study sample sizes, low success rates of HBV typing, and different study designs, the previously reported effects of HBV genotype and HBV mutations on the outcomes of HBV persistent infection have varied greatly.

Protein tyrosine phosphatase nonreceptor type 12 (PTPN12, also known as PTP‐PEST), is a member of the PTP (Protein tyrosine phosphatases) family 11. PTPN12 inhibits secondary T‐cell responses and is implicated in human autoimmunity 12, which may be either beneficial or detrimental to those infected with HBV 13. PTPN12 also regulates cell migration and cell–cell junctions 14, 15, and serves as an antagonist to tyrosine kinase signaling 16, 17, thereby plays an important role in tumor suppression. These findings highlight the importance of PTPN12 in the development of HBV‐related HCC.

*PTPN12* is located on chromosome 7q11.23. By utilizing bioinformatics analyses, we found that a single‐nucleotide polymorphism (SNP) around *PTPN12* may be an expression quantitative trait loci (eQTL) for *PTPN12* (http://www.regulomedb.org) 18, 19. Hence, we hypothesized that *PTPN12* eQTLs SNPs may contribute to risk of HBV infection and HCC. To test this hypothesis, we performed a case–control study to evaluate the effects of HBV genotype, mutations in the EnhII/BCP/PC region, *PTPN12* eQTLs SNPs, and their interactions on HCC risk.

## Methods

### Participants

This study was approved by the Institutional Review Board of Nanjing Medical University. The subjects' enrolment was described previously 20. To be brief, the HCC patients were recruited from January 2006 to May 2014 at the First Affiliated Hospital of Nanjing Medical University (Nanjing, China), the Nantong Tumour Hospital (Nantong, China), and the Qidong Liver Cancer Institute (Qidong, China) from central and southern Jiangsu Province, China. The diagnosis of HCC was confirmed by a pathological examination and/or an alpha‐fetoprotein elevation (>400 ng/ml) combined with an imaging examination. Since HCV infection is rare in Chinese populations, HCC patients with HCV infection were excluded. As a result, 1,507 HBV‐related HCC cases consented to participate in the study (535 cases from Nanjing, 522 cases from Nantong, and 450 cases from Qidong). The controls confirmed for being HBV persistent carriers from 2009 to 2010 were also from three cities in central and southern Jiangsu Province (48,417 subjects from Zhangjiagang, 43,563 subjects from Taixing, and 57,192 subjects from Danyang). HBV persistent carriers were those subjects who were positive for both hepatitis B surface antigen (HBsAg) and antibodies against the hepatitis B core antigen (anti‐HBc), but were negative for the HCV antibody (anti‐HCV) at the two visits. Approximately 2475 (5.11%), 3,413 (7.83%), and 5,587 (9.77%) HBV persistent carriers were identified from Zhangjiagang, Taixing, and Danyang, respectively. A total of 1560 cancer‐free controls were randomly selected from these three cities and were frequency matched to cases based on age and sex (510 controls from Zhangjiagang, 500 controls from Taixing, and 550 controls from Danyang). The demographic information of these selected controls, such as age and gender, was collected by face‐to‐face interviews.

### Serological testing

HBsAg, anti‐HBs, anti‐HBc, and anti‐HCV were detected by an enzyme‐linked immunosorbent assay (Kehua Bio‐Engineering Co., Ltd., Shanghai, China) in the serum, following the manufacturer's instructions as described previously 21.

### HBV mutation analysis

The HBV EnhII/BCP/PC region was amplified using nested PCR 22. The primers for the first and second round of the nested PCR were listed in our previous article 20. Direct DNA sequencing was carried out by using ABI PRISM BigDye sequencing kits and an ABI 3730 Genetic Analyser (Applied Biosystems, Foster City, CA). The HBV sequences were aligned and analyzed using (Mega 5.0, Arizona, U.S). After the alignment, the nucleotide with the highest frequency at each site in the HBV EnhII/BCP/PC region from the controls was termed as the wild‐type nucleotide. Nucleotide substitutions with the other three nucleotides or deletions at each site were termed as mutations. A site with combined mutation frequencies >10% from all the subjects was termed as a hotspot.

### Genotyping

The genomic DNA was extracted from the leukocyte pellets by traditional proteinase K digestion, followed by phenol–chloroform extraction and ethanol precipitation. Genotyping was performed using the TaqMan allelic discrimination assay on an ABI 7900 system (Applied Biosystems, La Jolla, CA). Detailed information for the primers and probes are shown in Table 1. Technicians were blinded to the status of cases and controls during genotyping; two blank (i.e., water) controls in each 384‐well format were used for quality control, and more than 10% of the samples were randomly selected to repeat, yielding a 100% concordant.

**Table 1:** Information of primers and probes for TaqMan allelic discrimination

Polymorphism	Sequence (5′–3′)	
rs11489585	Primers	F: TGAGAGTAATGACACACTCAATGTGGTA
		R: CTCTCCTTTTACGGCACTTCGT
	Probes	G: FAM‐ATGAGAAGTTCATTGTGG‐MGB
		A: HEX‐AGAAGTTCACTGTGGTTC‐MGB

### Statistical analysis

**Figure 1:**
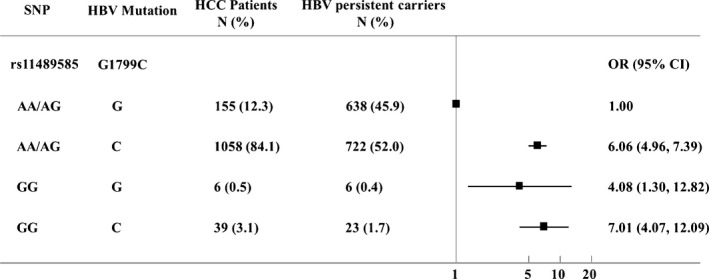
Crossover analysis of the SNP rs11489585‐HBV mutation interactions on HCC susceptibility. Crossover analysis suggested that the group of “C” at nt1799 with GG alleles (rs11489585) was associated with significantly increased risks (adjusted OR = 7.01, 95% CI = 4.07–12.09, *P* < 0.001) of chronic HBV infection, as compared with the group of “G” at nt1799 with AA/AG alleles (rs11489585).

The associations of the genotypes of the SNP with diseases risk were estimated by computing odds ratios (ORs) and their 95% confidence intervals (CIs) from logistic regression analyses. The heterogeneity of the associations between subgroups was assessed by the *χ*2 ‐based *Q* test. The multiplicative interactions of the SNP and all the HBV hotspot mutations with diseases risk were estimated by computing odds ratios (ORs) and their 95% CIs from logistic regression analyses (Table 4, Fig. 1). All the statistical analyses were performed with R software (version 2.13.0; The R Foundation for Statistical Computing, Vienna, Austria) and *P* ≤ 0.05 in a two‐sided test was considered statistically significant.

## Results

The demographic characteristics of the 1507 HBV‐positive HCC cases, 1560 HBV persistent carriers have been summarized previously 20. There were no significant differences in the distribution of age and gender between the two groups (*P* = 0.835 and 0.687, respectively, Table S1).

Among the subjects of this study, the HBV genotypes B, C, BC (coinfection), D and mutations in the EnhII/BCP/PC region were identified through nested multiplex PCR and sequencing as described previously. Of those 19 hotspot mutations, C1653T, T1674C/G, A1703G, G1719T, T1727A/G, T1753C, A1762T, G1764A, G1799C, G1899A, G1915A/C, and C1969T were significantly associated with an increased risk of HCC, whereas C1673T, A1726C, C1730G, and A1752G were significantly associated with a reduced risk of HCC, when adjusted for age and gender 20.

The genotype distributions of rs11489585 in the HCC patients and the HBV persistent carriers are described in Table 2. The observed genotype frequency for the SNP in the HBV persistent carriers were in Hardy–Weinberg equilibrium (*P *= 0.662). The logistic regression analyses in the recessive genetic model showed that the variant genotype GG of rs11489585 increased the host HCC risk compared with the HBV persistent carriers (adjusted OR = 2.03, 95% CI = 1.30–3.18). However, no heterogeneity was found between subgroups when we evaluated the effects of rs11489585 on HCC risk by stratifying on age, gender, or different HBV genotypes (Table 3).

**Table 2:** Association between rs11489585 and HBV‐related HCC susceptibility

Genotype	MAF	HWE	HCC patients	HBV persistent carriers	OR (95% CI)	*P* a
			(*n* = 1507)	(*n* = 1560)		
			*N* (%)	*N* (%)		
rs11489585	0.135	0.662				
AA			1085 (75.3)	1152 (75.0)	1	
AG			299 (20.8)	355 (23.1)	0.89 (0.75–1.06)	
GG			56 (3.9)	30 (2.0)	1.98 (1.26–3.11)	
Dominant					0.98 (0.83–1.16)	0.800
Recessive					2.03 (1.30–3.18)	0.002
Additive					1.06 (0.92–1.22)	0.410

a Logistic regression analyses adjusted for age and gender between HCC patients and HBV persistent carriers.

CI, confidence interval.

**Table 3:** Stratified analyses on rs11489585 with HCC susceptibility

Variables	HCC susceptibility (AA/AG/GG)			
	HCC Patients	HBV persistent carriers	OR (95% CI)	*P* a
Age				
≤53	675/174/38	646/189/14	2.01 (1.00–4.03)	0.385
>53	410/125/18	506/166/16	1.27 (0.59–2.73)	
Gender				
Male	875/247/47	933/281/24	1.60 (0.92–2.77)	0.737
Female	210/52/9	219/74/6	2.02 (0.58–7.00)	
HBV Genotype				
B‐related^b^	198/61/8	754/220/16	1.95 (0.82–4.62)	0.661
Non‐B^c^	862/232/47	382/127/14	1.54 (0.84–2.83)	

Logistic regression analyses adjusted for age, gender, and HBV genotype (excluded the stratified factor in each stratum) in recessive genetic model.

a *P*‐value for the heterogeneity test.

b B‐related genotypes including B, BC.

c Non‐B genotypes including C, D.

CI, confidence interval.

Multiplicative interactions of rs11489585 with all the HBV hotspot mutations were also evaluated (Table 4). We detected interaction between rs11489585 and the HBV mutation (*P* = 0.050 for G1799C). As shown in Figure 1, rs11489585 multiplicatively interacted with G1799C. Crossover analysis suggested that the group of “C” at nt1799 with GG alleles (rs11489585) was associated with significantly increased risks (adjusted OR = 7.01, 95% CI = 4.07–12.09, *P* < 0.001) of chronic HBV infection, as compared with the group of “G” at nt1799 with AA/AG alleles (rs11489585) (Fig. 1).

**Table 4:** Contributions of the interactions of rs11489585 with HBV mutations in the EnhII/BCP/PC region of HBV to HCC risk

SNP * Mutation	OR (95% CI)	*P*
rs11489585*nt1653	0.51 (0.16–1.66)	0.265
rs11489585*nt1673	1.14 (0.31–4.15)	0.848
rs11489585*nt1674	0.68 (0.21–2.18)	0.518
rs11489585*nt1703	4.00 (0.48–5.29)	0.943
rs11489585*nt1719	0.57 (0.21–1.58)	0.282
rs11489585*nt1726	2.44 (0.50–12.0)	0.272
rs11489585*nt1727	0.74 (0.20–2.72)	0.646
rs11489585*nt1730	1.79 (0.46–6.89)	0.398
rs11489585*nt1752	1.00 (0.95–2.31)	0.959
rs11489585*nt1753	0.43 (0.14–1.34)	0.146
rs11489585*nt1762	0.70 (0.19–2.62)	0.594
rs11489585*nt1764	0.39 (0.11–1.37)	0.141
rs11489585*nt1799	0.28 (0.08–1.00)	0.050
rs11489585*nt1846	0.74 (0.27–2.02)	0.555
rs11489585*nt1896	1.31 (0.51–3.42)	0.575
rs11489585*nt1899	0.47 (0.14–1.51)	0.203
rs11489585*nt1915	0.62 (0.16–2.48)	0.500
rs11489585*nt1969	0.82 (0.15–4.35)	0.815
rs11489585*nt1979	0.32 (0.09–1.14)	0.079

Logistic regression analyses adjusted for age and gender in the recessive genetic model.

## Discussion

The development of HCC is a multistage process, and HBV mutations gradually occur in the progression of chronic HBV infection 23. The HBV mutations are probably generated via an evolutionary process on two aspects: increased frequencies of the viral mutations and directional selection of the mutations by the host immune system 24. The HCC‐associated mutations often occur in the Enh II/BCP/precore 25. Numerous associations between HBV mutations and risk of HCC had been identified 26. A1762T, G1764A, and T1753C/A mutations in the BCP region, and G1613A and C1653T mutations in the Enh II region were reported more frequent in HCC patients 27. Our previous study had also found several novel HBV mutations associated with HBV‐related HCC, including A1752G, G1915A/C, and C1969T 20.

Previously, a case–control study, including 1108 HCC patients and 1628 HBV‐positive subjects without HCC, was conducted by Cao et al. to evaluate the effects of *HLA‐DP* polymorphisms, HBV EnhII/BCP/PC region mutations, and their interactions in HCC risk. They found that the interactions of rs9277535 AA with the T1674C/G or G1719T mutation in genotype C significantly decreased HCC risk 28. They also explored the interactions of *STAT3* SNPs with HBV mutation on HCC risk in 1021 HCC patients and 990 HBV‐positive subjects without HCC 29. Recently, our group also revealed significant interactions of *HLA‐DQ/DR* rs9272105 with both the HBV genotype and mutations (*P* < 0.05 for each) on account of the large sample size and the high detection rate. Still, more genes with potential interaction with HBV sequence variation need to be identified. Here, based on large sample size, we detected significant interactions of *PTPN12* eQTL SNP rs11489585 with HBV mutation, which may imply a potential biological significance for rs11489585.

Several previous studies have reported that PTPN12 may regulate the equilibrium of tyrosine phosphorylation and play a prominent role in tumor suppression 30, 31. *PTPN12* variants, such as Ile322 and Ala573, might be involved in regulating phosphatase activity and then be meaningful for human cancer 31. Also, PTPN12, inhibits secondary T‐cell responses and is implicated in human autoimmunity, which may have implications in those infected with HBV^13^. These evidences support the importance of PTPN12 in HBV‐related HCC.

Interestingly, though rs11489585 was an eQTL SNP for *PTPN12,* it indeed is located within a long noncoding RNA *APTR*. Long noncoding RNAs are emerging as key players in various fundamental biological processes 32. Abnormal lncRNAs expression can influence genes associated with hepatocarcinogenesis 33. Dysregulation of lncRNAs, including *H19*,* HEIH*,* MVIH*,* HULC*, and *MEG3*, has been identified in HCC 34, 35, 36. In some cases, the SNPs in lncRNAs may regulate the expression of localized lncRNAs and then affect the expression or function of nearby genes. Our previous published study had shown that three *ZNRD1* eQTL SNPs (rs9261204, rs6940552, and rs3757328) were associated with increased HCC risk. In our current study, the variant genotype GG of rs11489585 in *APTR* increased the host HCC risk in a large Han Chinese population.

In summary, this study, with a relative large population, showed that *PTPN12* eQTL SNP may interact with HBV mutation on HCC risk. Further studies with functional assays conducted in diverse populations are needed to validate and extend our findings.

## Conflict of Interest

None declared.

## Supporting information


**Table S1.** Distribution of selected demographic variables in HCC and HBV persistent carrier.
