# Supplementary material for: Hepatitis B virus mutations, expression quantitative trait loci for PTPN12, and their interactions in hepatocellular carcinoma
Source: Cancer Med. 2016 Apr 14;5(7):1687–93. doi: 10.1002/cam4.712 (PMC4944896; doi:10.1002/cam4.712)
Supplement: Supplementary file 1 — Table S1. Distribution of selected demographic variables in HCC and HBV persistent carrier. [file CAM4-5-1687-s001.doc]

| Supplementary Table S1. Distribution of selected demographic variables in HCC and HBV persistent carrier. | | | |
| --- | --- | --- | --- |
| Variable | HCC patients  (n =1507) N (%) | HBV persistent carriers  (n =1560) N (%) | *P* |
|
|
|
| Age | 51.1±10.7 | 51.2±10.0 | 0.835 |
| Gender |  |  |  |
| Male | 1221 (81.0) | 1255 (80.4) | 0.687 |
| Female | 286 (19.0) | 305 (19.6) |
| HBsAg | (+) | (+) | - |
| anti-HCV | (+) | (+) | - |
| anti-HCV | （-） | （-） | - |
